# Preferences for long‐acting Pre‐Exposure Prophylaxis (PrEP) for HIV prevention among South African youth: results of a discrete choice experiment

**DOI:** 10.1002/jia2.25528

**Published:** 2020-06-16

**Authors:** Alexandra M Minnis, Millicent Atujuna, Erica N Browne, Sheily Ndwayana, Miriam Hartmann, Siyaxolisa Sindelo, Nangamso Ngcwayi, Marco Boeri, Carol Mansfield, Linda‐Gail Bekker, Elizabeth T Montgomery

**Affiliations:** ^1^ Women’s Global Health Imperative RTI International San Francisco CA USA; ^2^ The Desmond Tutu HIV Centre University of Cape Town South Africa; ^3^ Health Preference Assessment RTI Health Solutions Belfast Ireland; ^4^ Health Solutions RTI International Research Triangle Park NC USA

**Keywords:** HIV prevention, long‐acting pre‐exposure prophylaxis, discrete choice experiment, acceptability, adolescent girls and young women, men who have sex with men

## Abstract

**Introduction:**

Existing biomedical HIV prevention options, though highly effective, present substantial adherence challenges. End‐user input on early‐stage design of new HIV prevention approaches is critical to yielding products that achieve high uptake and adherence. The iPrevent Study examined youths’ preferences for key attributes of long‐acting Pre‐Exposure Prophylaxis (PrEP), with a focus on characteristics pertinent to product delivery alongside key modifiable product attributes.

**Methods:**

A discrete choice experiment was conducted with female and male youth aged 18 to 24 in two high‐density communities in Cape Town, South Africa during the period July 2017 to January 2019. Sexually active, PrEP‐naïve youth were recruited using population‐based sampling; targeted sampling was used to enrol men who have sex with men (MSM). In a series of nine questions, participants were asked to choose between two hypothetical products composed of five attributes (form, dosing frequency, access, pain, insertion site). We used a random‐parameters logit model to estimate preference weights and trade‐offs among product alternatives. We examined differences across three subgroups: females, men who have sex with only women (MSW) and MSM.

**Results:**

A total of 807 participants (401 female) were enrolled with a median age of 21 years. Males included 190 MSM. Most youth had tested for HIV (95%) and reported being HIV‐negative (91%). Across all groups, duration of effectiveness was the most important attribute, with strong preference for less frequent dosing. Injections were favoured over implants, though these preferences were strongest for females and MSM. Females preferred a product offered at a health clinic and disliked pharmacy access; all groups preferred the arm as the insertion site. Youth were willing to trade their preferred product form for longer duration.

**Conclusions:**

Youth indicated strong preferences for longer duration products. Each attribute nonetheless influenced preferences, offering insight into trade‐offs that inform long‐acting PrEP development.

## 1 INTRODUCTION

End‐user input on early stage design of new HIV prevention approaches is critical to yielding products that achieve high uptake and adherence. As evidenced by multiple clinical trials, and, more recently, demonstration projects involving oral pre‐exposure prophylaxis (PrEP), existing prevention options, though highly effective, present substantial adherence challenges tied to dosing burden, product design and access and contextual factors that inhibit consistent and correct use [1, 2, 3, 4]. In sub‐Saharan African studies, biomarker assessments of daily oral PrEP and monthly vaginal ring use indicate that adherence was lowest among youth [3], highlighting the need for targeted and contextually relevant adherence interventions [5]. Long‐acting PrEP, including injectables and implants, overcome several of these barriers as they offer greater discreetness, require less frequent clinic visits and reduce adherence burden; thereby, addressing an important need in the choice continuum of options for HIV prevention.

Youth constitute a critical end‐user group that experiences disproportionately high rates of HIV and whose prevention needs are shaped by distinct social environments, interpersonal relationships and neurocognitive developmental stages [6]. The dynamic developmental period of adolescence and young adulthood influences partnerships, priorities and decision making regarding HIV prevention [7, 8]. In South Africa, 8.5% of youth aged 15 to 24 are HIV positive, and two‐thirds of new HIV infections occur in young women [9]. Young women additionally face gender inequalities and gender‐based violence that contribute to their heightened risk [10]. Although HIV prevalence peaks later in adulthood for males overall, young men who have sex with men (MSM) constitute a key population for HIV prevention in South Africa with an estimated prevalence of 26.8% [9]. Engaging youth to inform the design of future HIV prevention products is a priority, then, to achieving and sustaining HIV prevention objectives.

Discrete choice experiments (DCEs) offer an efficient and robust approach to assess product preferences by examining the trade‐offs individuals make when asked to choose between alternative product designs [11]. DCEs measure stated preference, that is, the anticipated choice individuals would make if presented with an opportunity to actually choose between real products. Though DCE choices are hypothetical, this methodology is particularly well‐suited to informing the design of prevention products or other interventions when actual options do not exist. In HIV prevention, DCEs have been used increasingly to inform intervention features [12] and generate insights for the development of novel biomedical HIV prevention delivery forms, including multipurpose prevention technologies that prevent HIV and pregnancy [13, 14, 15, 16].

The iPrevent Study was designed to engage youth to inform the HIV prevention product pipeline. The specific objective of this phase of iPrevent was to examine youth’s preferences among key attributes of long‐acting PrEP. While there are multiple long‐acting PrEP delivery forms in the development, research and regulatory review pipeline, including microneedle patches, passive antibody transfer (broadly neutralizing antibodies [bnAbs]), long‐acting oral PrEP and vaginal rings [17], iPrevent focused on two systemic delivery forms – implants and injectables – that share core attributes allowing for alignment in the DCE design and that, at the time we designed the study, were among the furthest along in the development pipeline, making them good candidates for this study. Through a DCE, we integrated attributes pertinent to product delivery alongside characteristics of the products themselves. We assessed whether preferences differed between female and male youth, and, among males, between men who have sex with only women (MSW) and MSM. Finally, we compared trade‐offs among the most influential attributes to evaluate what would influence youth to choose one product design over another.

## 2 METHODS

### 2.1 Setting

iPrevent was conducted in two informal peri‐urban communities, Nyanga and Masiphumelele, near Cape Town, South Africa. Established as residential areas for black South Africans during apartheid [18], the communities are characterized by extreme overcrowding, unemployment and endemic violence. Both suffer from a generalized HIV epidemic with prevalence currently 13.1% [19, 20]. The Desmond Tutu HIV Foundation (DTHF), the South African‐based partner in this research, has well‐established research sites and community advisory boards (CABs) in both locations.

### 2.2 Sampling and recruitment

Eligible participants were female and male youth aged 18 to 24 who had not participated in a biomedical HIV prevention trial of a PrEP product (i.e. vaginal gel, vaginal ring, oral tablet or injection). This exclusion criterion ensured that all participants were “product naïve” in their evaluation of long‐acting PrEP attribute preferences. HIV status was not an eligibility criterion as we designed the study to be conducted in community settings where we did not have the resources to test participants for HIV as part of screening procedures. We did collect HIV status via self‐report. The target enrolment was 400 females and 400 males (approximately half MSM). Sample size calculations represent a challenge in choice experiment because the models used to estimate preference weights simultaneously estimate multiple coefficients. Proposed approaches to calculating the sample size for a discrete‐choice experiment rarely address the issue of how to determine the minimum sample size required to provide statistical power for hypothesis tests on specific coefficients, or on specific subgroups. Some approaches require specification of expected relative sizes of preference weights to be estimated [19, 20]; however, prior expectations of effect sizes are often not known. Our sample size was established based on the DCE design parameters (i.e. number of choice tasks, alternatives per task and attribute levels) and pre‐specified sub‐groups analyses [21]. Empirical studies suggest that for a DCE with six to eight attributes and three levels per attribute, a sample size of 250 to 350 respondents (each of whom are presented with 8 to 10 choice questions) is required to provide sufficient information to identify preferences with acceptable precision [22]. Subgroup analysis often can be conducted using smaller sample sizes (approximately 150 respondents) by estimating a model using the full sample and interaction terms to identify differences between subgroups. Thus, our design, which included five attributes, provided additional power, even for smaller subgroups, such as MSM who were a priori of particular interest.

We recruited a general population‐based sample of youth (400 female, 200 male) during the period July 2017 to December 2018. Recruitment and pre‐screening occurred in the community (at residential plots for the 600 participants comprising the population‐based sample; for the MSM sample, varied community locations) while study visits took place at community‐based research sites established by DTHF. We conducted the sampling and recruitment specifically for the iPrevent study. In Nyanga we designed a two‐stage sampling procedure involving selection of primary and secondary sampling units (SSUs) that constituted the recruitment zones within a two‐kilometre radius from DTHF’s primary research site. Within SSUs, recruitment staff sampled every nth plot (i.e. fourth, fifth or sixth) based on the housing density of the SSU as determined by City of Cape Town census maps, to achieve an approximately equal probability of selection. Recruitment focused on plots, rather than households, to ensure youth residing in all dwellings in a selected plot (backyard and main house) were enumerated and, if deemed eligible, recruited into the study. Nyanga experienced high levels of violence during the recruitment period, including murders that prompted substantial conflict, road blockades, fires and threats to safety preventing staff from accessing the areas [23]. Therefore, in consultation with the CAB and study team members who resided in the community, we identified wards within the catchment area that were relatively more safe and sampled replacement SSUs from within those areas. In Masiphumelele, we drew on an existing household census conducted by DTHF for another study. Owing to the rapid emergence and expansion of informal settlements, we additionally sampled plots where dwellings did not align with the census using the methods designed for Nyanga.

To enrol a population of 200 young MSM, we used respondent‐driven sampling (RDS) supplemented by convenience sampling. Working with DTHF’s network of “safe spaces” in four high‐density communities, we conducted formative design research and identified initial respondents (“seeds”) to whom we provided up to three recruitment coupons for distribution within their social networks. Participants (including seeds) received modest incentives for all recruited participants to whom they gave coupons and who came for screening. We aimed to build recruitment chains using standard RDS methodology and tailored best‐practices to our setting [24, 25]. RDS proved extremely challenging to implement owing to difficulty in establishing referral chains that persisted for multiple waves. Therefore, we modified our sampling procedures in the following ways: adding seeds (increased from an initial four to thirteen total seeds), increasing the number of referral coupons distributed (up to 5), offering free wifi access at the site, encouraging electronic distribution of referral coupons within a social network (via WhatsApp), and allowing PrEP‐experienced youth to enrol since PrEP had become increasingly available to MSM in Cape Town over the study period. Ultimately, we implemented targeted convenience sampling when it became evident after 107 coupons returned, despite these efforts, that RDS would not yield a representative sample of MSM [26].

The ethical review committee at the University of Cape Town (HREC 751/2015) approved the study. All participants provided informed consent.

### 2.3 DCE development

Product attributes and levels presented in the DCE were selected based on a priori identification of key attributes, formative research, expert consultations, youth CAB feedback and pre‐testing. Formative research consisted of 50 in‐depth interviews with youth aged 18 to 24 who had prior experience with PrEP products as participants in clinical trials [27]. In addition, we conducted six focus group discussions with PrEP‐naïve youth, purposively recruiting females with contraceptive implant experience [28]. IDIs and FGDs addressed product features that affected adherence and ideal product attributes, informing both attribute selection and descriptions. We solicited input from product developers (approximately six teams from research organizations and pharmaceutical companies, purposively selected among those known to be developing long‐acting platforms) on long‐acting PrEP characteristics and delivery that this research could inform. Individual attribute descriptions and accompanying images were pre‐tested through an iterative process of youth CAB feedback and augmented by three rounds of formal cognitive testing with 20 youth recruited specifically for this phase. During pretests, we examined the clarity and cultural relevance of attribute descriptions and images. We finalized the attributes and levels included in the design to ensure they aligned with injectable and implant‐based products in pre‐clinical development and/or clinical trials. While all combinations of attribute levels are not reflected in current products in development, they were deemed potentially feasible targets based on product developer consultation and plausible to participants as ascertained through cognitive testing. Examination of how optimization of one attribute may affect acceptance of a less preferred level of another attribute is both a methodological strength of DCEs and reflects “real world” choices where it may not be possible to optimize all characteristics of a given prevention tool.

### 2.4 Study design

**Figure 1:**
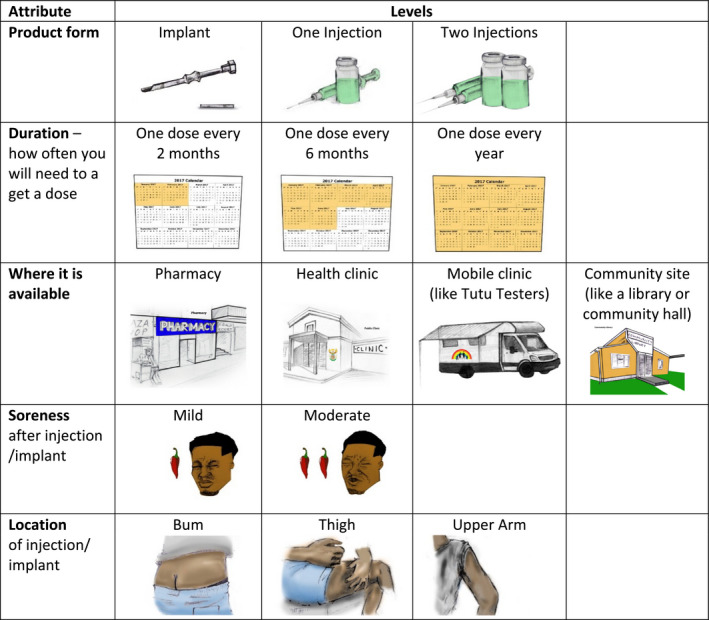
**Characteristics of an HIV prevention product that comprised the discrete choice experiment survey.** iPrevent Study, Cape Town, South Africa, 2017 to 2019.

Products were characterized by five attributes, each with two to four levels (Figure 1). Attributes included: product form (injection, implant); dosing frequency (two, six or twelve months); where to obtain the product (clinic, pharmacy, community distribution, mobile clinic – all models for current HIV prevention and contraceptive service delivery); pain involved with injection or insertion (mild, moderate) and delivery location on the body (arm, buttock, thigh). Each DCE choice question presented two hypothetical products composed of the five attributes. These pairs were constructed using an D‐optimal main‐effects experimental design [21, 29]; each question required respondents to make trade‐offs among the attributes, with trade‐offs varying systematically across questions. The experimental design included 36 choice questions that were divided equally into four blocks. Respondents were randomly assigned to one block, with question order randomized within block. A block, therefore, consisted of nine choice questions, each question presenting a choice between Product A and Product B. We included images for each attribute level to assist cognitive processing and provide visual aids for lower literacy respondents.

To prime participants for decision making regarding long‐acting PrEP, an educational video was shown prior to initiating the DCE. The video explained PrEP, communicated that scientists are developing new methods for delivering PrEP, and encouraged youth to “add their voice” to inform the next generation of HIV prevention products. We held pre‐ and post‐production meetings with DTHF’s youth CAB to review the script, setting (a minibus taxi, which is widely used for informal public transportation) and tone.

### 2.5 Data collection

Participants completed interviews on a tablet computer, with assistance from a research interviewer. The survey first introduced each attribute individually with both visual and narrative descriptions. Interviewers guided participants through each attribute description, pausing to ensure they understood each one before presenting the next. Participants were then presented with nine DCE choice questions, each one a unique choice (see Figure S1 for example). Following each choice, participants were asked: “If the product you just chose was available, do you think you would actually use it?” [yes/no]. We also measured participant socio‐demographic factors, HIV risk perception and sexual behaviours.

### 2.6 Analysis

A random‐parameters logit (RPL) model was used to estimate preference weights for each attribute level. RPL models are commonly used for analysis of preference data, as they can account for participant heterogeneity by estimating a normal distribution for each attribute level [30, 31]. All attribute levels were categorical effects‐coded variables. With this style of coding, the omitted level is estimated from the negative sum of all other levels included in the model [32]. Therefore, the estimates for each level represent the preference for that level relative to the mean attribute effect (as opposed to the reference group in dummy coding). Since data were collected by subgroup (females, MSW and MSM) using different methods and from two different communities, we first tested for differences in preference and scale following a procedure outlined by Swait and Louviere [33]. The final RPL model included fixed interaction terms between attribute levels and binary indicators of subgroups, with females as the referent category. Wald tests were used postestimation to test for differences in attribute level preferences between subgroups. We conducted a sensitivity analysis excluding those youth who were HIV positive per self‐report (N = 42, 5%). *p* < 0.05 were considered statistically significant. All analyses were performed using Stata 15.0 (StataCorp, College Station, TA, USA).

We present RPL results graphically, displaying the mean preference‐weight estimates for each attribute relative to the mean attribute effect, normalized around zero, with 95% confidence intervals. Positive weights indicate greater preference and negative weights indicate less preference relative to the other levels evaluated. The relative importance of an attribute overall is depicted by the vertical distance between the most‐preferred and least‐preferred levels, that is the difference between the largest preference weight and the smallest preference weight. Finally, we used preference weights to examine trade‐offs youth were willing to make between pairs of attributes. We calculated the minimum acceptable dosage (in months) that a product would need to offer for participants to be willing to trade one attribute level for another (e.g. from single to dual injections) by calculating the average difference in utility per month using the dosing frequency effect‐coded parameter estimates and assuming linearity between the levels.

## 3 RESULTS

Overall, 807 youth completed the DCE survey (50% female). Nearly half (47%) of male participants were MSM. The median age was 21 years (interquartile range: 19 to 22). Participants’ socio‐demographic and behavioural characteristics, overall and by sex‐behaviour subgroups, are presented in Table 1. In general, most youth were unemployed (70%), currently had a primary partner (74%) and had >2 sex partners in their lifetime (81%). Nearly all (95%) had ever been tested for HIV; 42 (5%) were HIV‐positive. Several behavioural characteristics differed between subgroups, with all men reporting a higher median number of lifetime partners and more partners in the past three months. Among females, 77% had ever used injectable contraceptives and 16% had ever used contraceptive implants. The 608 youth enrolled through population‐based sampling were identified based on 720 screened from among 1176 individuals identified from plots with one or more potential participant. Reasons for non‐enrolment among those screened included: refusal (n = 81); screening failures (n = 31); current PrEP use (n = 15); and not age eligible (n = 16).

**Table 1:** Socio‐demographic and behavioural characteristics of participants. iPrevent Study, Cape Town, South Africa, 2017 to 2019

	Overall	Female	Male: MSW	Male: MSM
	N (%)	N (%)	N (%)	N (%)
Total	807 (100)	401 (100)	216 (100)	190 (100)
Socio‐demographic factors				
Age, years – median (IQR)	21 (19 to 22)	21 (19 to 22)	21 (19 to 22)	20 (19 to 22)
Less than secondary school	340 (42)	179 (45)	104 (48)	57 (30)
Currently in school	318 (39)	123 (31)	85 (39)	110 (58)
Employment				
Formal	120 (15)	55 (14)	34 (16)	31 (16)
Informal	123 (15)	51 (13)	50 (23)	22 (12)
None	564 (70)	295 (74)	132 (61)	137 (72)
Food insecurity (past month)^a^	210 (26)	91 (23)	50 (23)	69 (36)
Parity >0 (or fathered a child)	225 (28)	182 (45)	35 (16)	8 (4)
Household crowding^b^	172 (21)	117 (29)	45 (21)	10 (5)
Behavioural factors				
Lifetime number of sexual partners ‐ median (IQR)	4 (3 to 6)	3 (2 to 4)	6 (4 to 10)	5 (4 to 10)
Has primary partner	594 (74)	327 (82)	161 (75)	106 (56)
Multiple partners past three months	208 (26)	31 (8)	88 (41)	89 (47)
Ever used condoms	758 (94)	375 (94)	199 (92)	184 (97)
Condom use at last sex	487 (60)	237 (59)	119 (55)	131 (69)
HIV testing and status				
Ever tested for HIV	765 (95)	392 (98)	193 (89)	180 (95)
HIV status				
Negative	701 (87)	360 (90)	175 (81)	166 (87)
Positive	42 (5)	27 (7)	4 (2)	11 (6)
Unknown	64 (8)	14 (4)	37 (17)	13 (7)
Community of residence				
Masiphumelele	345 (43)	248 (62)	91 (42)	6 (3)
Nyanga	308 (38)	153 (38)	115 (53)	40 (21)
Other	154 (19)	0 (0)	10 (5)	144 (76)
Contraceptive method use (ever)				
Injectable		310 (77)		
Implant		62 (16)		
Oral contraceptive pills		70 (18)		

MSM, men who have sex with men; MSW, men who have sex with women only.

a “Sometimes” or “often” worried about not having enough food

b more than two persons per room.

### 3.1 Long‐acting HIV prevention product preferences

**Figure 2:**
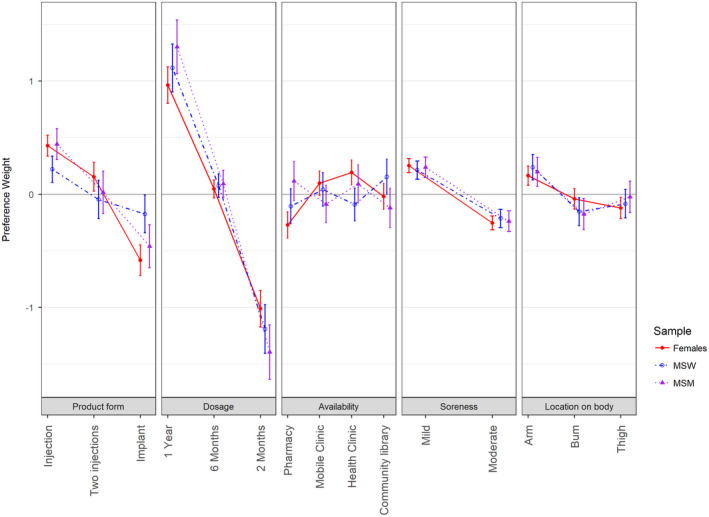
**Normalized preference weights with 95% confidence intervals, by sample subgroup.** MSM, Men who have sex with men; MSW, Men who have sex with women only.

Following the test outlined by Swait and Louviere [33], we found no difference in preference or scale by recruitment strategy or community. Preferences, however, were found to differ significantly between females, MSW and MSM; therefore, preference weights for each attribute level are presented (Table 2) and depicted (Figure 2) by subgroup.

**Table 2:** Normalized preference weights for long‐acting HIV prevention product attributes estimated from a RPL model (N = 807)

	Female		MSW		MSM	
	Coef.	SE (95% CI)	Coef.	SE (95% CI)	Coef.	SE (95% CI)
Product form						
Injection	0.43	0.05 (0.34, 0.52)	0.22	0.06 (0.10, 0.33)	0.45	0.07 (0.31, 0.58)
Two injections	0.15	0.06 (0.03, 0.28)	−0.05	0.09 (−0.21, 0.12)	0.01	0.09 (−0.18, 0.20)
Implant	−0.58	0.07 (−0.71, −0.44)	−0.17	0.08 (−0.34, −0.01)	−0.46	0.10 (−0.65, −0.27)
Dosage						
2 months	−1.01	0.08 (−1.17, −0.85)	−1.18	0.11 (−1.39, −0.97)	−1.41	0.12 (−1.65, −1.17)
6 months	0.05	0.04 (−0.03, 0.13)	0.07	0.05 (−0.03, 0.18)	0.10	0.06 (−0.02, 0.22)
1 year	0.96	0.08 (0.80, 1.12)	1.11	0.11 (0.90, 1.32)	1.31	0.12 (1.07, 1.55)
Where it is available						
Pharmacy	−0.27	0.06 (−0.39, −0.15)	−0.11	0.08 (−0.26, 0.05)	0.12	0.09 (−0.05, 0.29)
Mobile clinic	0.10	0.05 (−0.01, 0.20)	0.04	0.07 (−0.10, 0.19)	−0.09	0.08 (−0.26, 0.07)
Health clinic	0.19	0.06 (0.08, 0.30)	−0.09	0.07 (−0.23, 0.05)	0.10	0.09 (−0.07, 0.27)
Community site	−0.02	0.06 (−0.13, 0.10)	0.15	0.08 (0.00, 0.31)	−0.13	0.09 (−0.30, 0.05)
Soreness						
Mild	0.25	0.03 (0.19, 0.31)	0.21	0.04 (0.13, 0.29)	0.24	0.05 (0.15, 0.33)
Moderate	−0.25	0.03 (−0.31, −0.19)	−0.21	0.04 (−0.29, −0.13)	−0.24	0.05 (−0.33, −0.15)
Location on body						
Arm	0.16	0.04 (0.08, 0.25)	0.24	0.06 (0.13, 0.35)	0.21	0.07 (0.08, 0.34)
Bum	−0.04	0.05 (−0.13, 0.05)	−0.15	0.06 (−0.28, −0.03)	−0.18	0.07 (−0.32, −0.04)
Thigh	−0.12	0.05 (−0.21, −0.03)	−0.08	0.06 (−0.21, 0.04)	−0.03	0.07 (−0.17, 0.11)

Fixed interaction terms between attribute levels and binary indicators for MSM and MSW were included in the model. Mean preference weights were calculated postestimation using linear combinations of coefficients. CI, confidence interval; Coef, coefficient; MSM, men who have sex with men; MSW, men who have sex with women only; RPL, random‐parameters logit; SE, standard error.

All five attributes in the DCE influenced preferences; however, two – dosing frequency and product form – exerted the greatest effects on choice. Dosing frequency constituted the most important attribute influencing preference for a long‐acting HIV‐prevention product. All three subgroups had strong preference for a product with a one‐year duration over two months (*p* < 0.001). MSW placed the most importance on dosing frequency, with it being five times more important than any other attribute. Within dosing frequency, MSM expressed stronger preference for a longer duration product than females, evidenced by a significantly greater difference in preference between a one‐year duration and two‐month duration product, *p* = 0.002).

Product delivery form was the second most important, with injections preferred over implants. However, females and MSM had stronger opinions about form; both had greater preference for a single injection over an implant compared to MSW (*p* ≤ 0.004). Although more modest in magnitude, females and MSW also expressed more preference for two injections compared with implants (*p* ≤ 0.009), whereas MSW had no difference in preference (*p* = 0.43).

Where the product is available and insertion/injection location were relatively less important; however, youth had some opinions about location alternatives. Females preferred using a product that was offered at a health clinic over accessing it at a pharmacy (*p* < 0.001). Among males, MSW had somewhat more preference for availability at a community location compared with a pharmacy and health clinic, whereas MSM held opposite views with pharmacy or health clinic preferred over a community location (*p* = 0.01). All youth preferred product insertion in the arm (*p* < 0.001). Females disliked insertion in the thigh and both MSW and MSM disliked insertion on the buttocks (*p* = 0.01).

Overall, youth indicated strong interest in using a long‐acting HIV prevention product; for 96% of the choice tasks (7032/7263), respondents stated they would be willing to use their chosen product if available. Across the sample, 118 participants (15%) opted out of at least one choice question (i.e. stated they would not use their chosen product); MSM were significantly more likely to opt‐out (23%) than MSW (14%) and females (11%). Only 11 participants (1%) opted‐out of five or more choices, and only one opted‐out of all nine choices. In sensitivity analyses in which we excluded the 5% of youth who were HIV positive, per self‐report, we found no differences in preference estimates. Furthermore, HIV positivity was not associated with opting out of chosen products.

### 3.2 Trade‐offs between attributes

Given the relative importance of duration and delivery form, the trade‐offs analysis focused, first and foremost, on the relationship between these two attributes. The value or utility gained from increasing the dosing frequency from two to six months was greater than increasing from 6‐months to 12‐months (*p* < 0.001). Therefore, monthly utility values were calculated separately when incrementing between two‐ to six‐ and six months or greater. Table 3 presents, by subgroup, the estimated trade‐offs youth were willing to make, in terms of months of dosing frequency, to move from an injectable to an implant. On average, females were willing to accept an implant if it provided 4.8 months of protection when compared with a 2‐month dual injection (95% CI: 3.7, 5.8 months) and 5.8 months when compared with a 2‐month single injection (95% CI: 4.9, 6.7). If injectables are dosed every six months, females were willing to accept an implant only if it provides 10.8 months of protection compared to a dual injection (95% CI: 9.0, 12.6) and 12.6 months compared to a single injection (95% CI: 11.0, 14.2). Because, on average, males valued duration more than product form, they did not require as many months of additional protection to accept an implant.

**Table 3:** The minimum acceptable implant dosing frequency required (in months) for youth to be willing to trade a 2‐ or 6‐ month injectable (dual or single) for an implant, by subgroup

	2‐month Dual injection		2‐month Single injection		6‐month Dual injection		6‐month Single injection	
	Implant (months)	95% CI	Implant (months)	95% CI	Implant (months)	95% CI	Implant (months)	95% CI
Females	4.8	(3.7, 5.8)	5.8	(4.9, 6.7)	10.8	(9.0, 12.6)	12.6	(11.0, 14.2)
MSW	2.4	(1.4, 3.4)	3.2	(2.5, 4.0)	6.7	(4.9, 8.6)	8.3	(6.9, 9.7)
MSM	3.2	(2.3, 4.2)	4.4	(3.6, 5.2)	8.3	(6.6, 10.1)	10.5	(9.0, 12.0)

CI, confidence interval; MSM, Men who have sex with men; MSW, Men who have sex with women only.

In addition, we considered trade‐offs between access location and duration for females given significant differences in preferences. If a clinic, the preferred location, offered a 2‐month long‐acting PrEP product, females would be willing to go to a pharmacy if the pharmacy offered a product dosed every 3.8 months (95% CI 3.0, 5.5); if the clinic offered a six‐month long‐acting PrEP product, they would be willing to go to the pharmacy if it offered a product dosed every 9.0 months (95% CI 7.7, 10.4).

## 4 DISCUSSION

This study of preferences for long‐acting PrEP among a population‐based sample of youth in Cape Town, South Africa, supplemented by a targeted sample of MSM, highlighted the importance of dosing frequency and product form, with preference for less frequent dosing and for injectables over implants. These findings extend those from the formative qualitative phase of our research that underscored the salience of “invisibility” to HIV prevention decisions, with youth expressing interest in products that require minimal burden, have sustained effectiveness and can be used without others knowing [27]. Stated preference data derived from other DCE studies consistently find that choice of highly effective products predominates consideration of other attributes [13, 15]. In this study, we chose not to include efficacy under the assumption that long‐acting products would generally confer high efficacy and, instead, directed attention to other modifiable attributes to understand those most influential to product preferences.

Despite dosing frequency constituting the most important attribute overall, it was relatively more important to MSW than to females and MSM. Product form played a secondary, but nonetheless prominent role in product choice. In general, females expressed more preference for injections (single and dual) and greater dislike of implants, compared with MSW, likely reflecting females’ familiarity with and widespread use of contraceptive injections. Although disliked over injections, MSW regarded an implant as more favourable than did MSM and, as illustrated by the trade‐offs analysis, with relatively modest increases in duration of effectiveness, were willing to trade an injection for an implant to achieve less frequent dosing. Females’ and MSM’s stronger preference for injections over implants may reflect a greater concern for discreteness in selection of an HIV prevention product, with the greater invisibility offered by the injection being particularly appealing. In addition to aligning with iPrevent’s qualitative findings [27], other research has demonstrated stigma and partner barriers to PrEP access and use, underscoring the appeal of a product that cannot be detected by partners and other household members [34, 35]. The perceived need for an “invisible product” may be less critical for heterosexual male youth.

The findings regarding preferences for where the product is available and administration location on the body highlight important differences by subgroup, with implications relevant to next‐generation product design and implementation work. Females preferred to obtain products in clinic locations, which may reflect a comfort with familiar, trusted locations where contraceptives and services are obtained at no cost. Despite considerable evidence regarding systems‐level barriers to youth accessing sexual health services at clinics [36], the co‐location of HIV prevention and contraceptive services may also have been perceived as efficient and convenient, which also reflects an important commitment toward integrated services as highlighted by Evidence for Contraceptive Options in HIV Outcomes (ECHO) trial findings [37]. Alternative delivery of contraceptives, such as self‐administration of injectables [38], may shift expectations for availability of similarly delivered HIV prevention. The dislike of pharmacy access likely reflects the expectation that this would require costs associated with paying for medicines out‐of‐pocket, as opposed to public clinics where no costs are incurred. This may be particularly salient for youth who lack financial independence. In contrast, MSW’s preference for product access in community locations, mirrors their less frequent engagement with health services, which has prompted community‐based distribution of HIV self‐testing [39]. Males’ dislike of product administration in the buttocks, compared with arm, may warrant consideration in the development of future injectable products as, though existing cabotegravir injections are administered in the buttocks, other drug formulations may allow for alternative administration locations on the body which could also align with products more readily administered in community‐based settings.

The clear subgroup differences in preference echoes findings from other preference studies conducted in the United States and multiple African countries, that highlight differences in preference across PrEP delivery forms between males and females. HPTN 077, for example, evaluated injectable cabotegravir safety and acceptability among HIV negative men and women and found within the U.S. that preferences for injectable PrEP were higher among men than women and an overall higher preference for injectable products among participants at African sites [40]. While other studies offer insight regarding preferences across delivery forms (e.g. implantable vs. injectable PrEP vs. daily oral PrEP), they have often been conducted in a single population (e.g. MSM) or in distinct geographic areas, making direct comparison of findings challenging while also emphasizing variation in preferred delivery forms across populations (e.g. [41]). Our previous work in TRIO and Quatro, two user‐preference studies conducted with women aged 18 to 30 in Kenya, South Africa and Zimbabwe, underscored preference differences within young adult women by geography, educational level and other contextual factors [13, 42, 43].

Several limitations of iPrevent should be considered when interpreting results. First and foremost, DCEs measure only hypothetical acceptability of product features rather than preferences after use of actual or placebo products. This approach is an efficient and methodologically rigorous strategy to solicit end‐user input to inform product development when actual products do not yet exist. Research exploring the external validity of DCEs – the degree to which stated preferences align with actual behaviour – has suggested positive predictive values to be reasonably high in accurately predicting choices, whereas negative predictive values have been more moderate [44, 45]. Second, the preferences and trade‐offs evaluated are based only on those attributes included in the design. Nonetheless, we focused on modifiable attributes that were identified through extensive formative research. Third, the lack of an opt‐out alternative in the DCE does not allow us to measure actual demand for the products and means we estimated choice probabilities conditional on use of a new product. Fourth, despite preferences for injectable products, the current cabotegravir intramuscular injection regimen (one injection every eight weeks) evaluated in two ongoing HIV prevention clinical studies (HPTN 083 [MSM and transgender women] and 084 [women]) requires daily oral PrEP use for one year after the last injection to prevent drug‐resistant infection if participants acquire HIV during this period [46]. This two‐product regimen to cover the pharmacokinetic tail was not evaluated in iPrevent but could certainly influence product choice. Fifth, our challenges in recruiting young MSM using RDS required that we rely on convenience sampling methods for nearly half of our MSM sample, limiting generalization. Finally, some of the differences between subgroups could be caused by scale (i.e. variability in preference) instead of preference heterogeneity. The Swait and Louviere test [33] highlighted that conditional on recruitment strategy, males and females have different preferences, which implies that preference and scale heterogeneity are confounded.

## 5 CONCLUSION

This DCE, conducted with a rigorous, population‐based design in two peri‐urban Cape Town communities, identified youth’s preferences in considering long‐acting injectable and implantable PrEP. While, overall, dosing frequency and product form constituted the most influential attributes, all five attributes shaped preferences. Comparison of preferences between female, MSW and MSM subgroups highlighted important differences informative to product developers and implementation work. Youth exhibited a keen interest in long‐acting PrEP, highlighting the potential for these products to fill important gaps in the existing toolbox and expand choice.

## COMPETING INTERESTS

The authors have no conflicts of interest pertinent to this research to declare.

## AUTHORS’ CONTRIBUTIONS

AM, MA and EM led study design and implementation, defined the manuscript scope and guided analysis and interpretation of results. AM led manuscript writing and development. EB conducted data analysis, synthesized results and contributed to manuscript writing. NN led field implementation and contributed to interpretation of results. MH contributed to study implementation, presentation and interpretation of results and manuscript review. SS and SN provided leadership in data collection and informed interpretation of results. MB and CM provided scientific oversight for the DCE design, analysis and interpretation of results. LGB contributed to conceptualization of the study, provided scientific oversight and contributed to interpretation of results and manuscript review. All authors approved the final manuscript as submitted and agree to be accountable for all aspects of the work.

## Supporting information


**Figure S1.** Example choice question from discrete choice experiment survey, iPrevent Study, Cape Town, South Africa, 2017 to 2019. Participants were asked to choose which HIV prevention product they would prefer to use.
