# Supplementary material for: Preferences for long‐acting Pre‐Exposure Prophylaxis (PrEP) for HIV prevention among South African youth: results of a discrete choice experiment
Source: J Int AIDS Soc. 2020 Jun 16;23(6):e25528. doi: 10.1002/jia2.25528 (PMC7297460; doi:10.1002/jia2.25528)

Supplemental Figure 1. Example choice question from discrete choice experiment survey, iPrevent Study, Cape Town, South Africa, 2017-2019. Participants were asked to choose which HIV prevention product they would prefer to use.


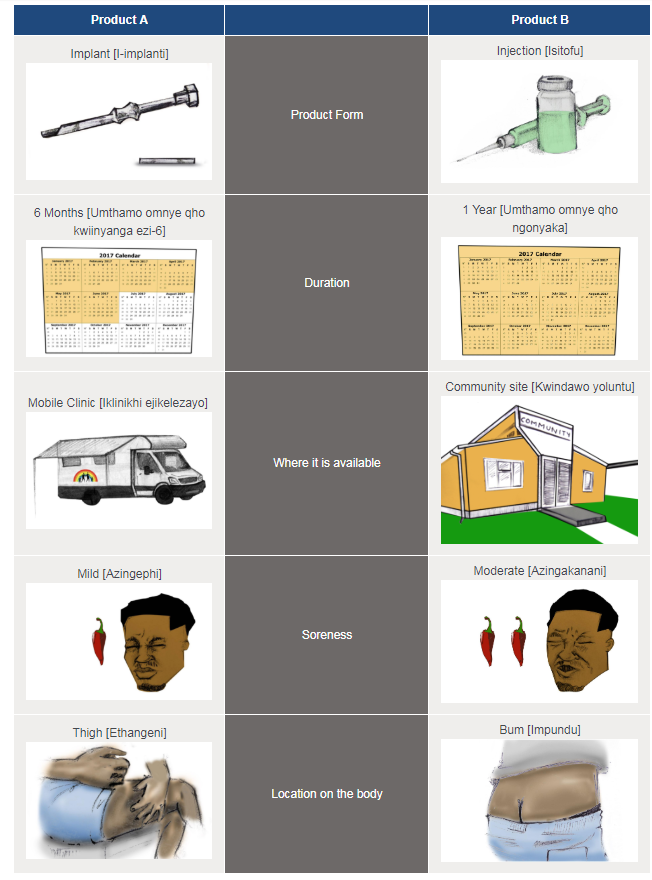

Supplement: Supplementary file 1 — Figure S1. Example choice question from discrete choice experiment survey, iPrevent Study, Cape Town, South Africa, 2017 to 2019. Participants were asked to choose which HIV prevention product they would prefer to use. [file JIA2-23-e25528-s001.docx]
